# Prioritization of Mycotoxins Based on Their Genotoxic Potential with an In Silico-In Vitro Strategy

**DOI:** 10.3390/toxins13100734

**Published:** 2021-10-19

**Authors:** Maria Alonso-Jauregui, María Font, Elena González-Peñas, Adela López de Cerain, Ariane Vettorazzi

**Affiliations:** 1 Department of Pharmacology and Toxicology, Research Group MITOX, School of Pharmacy and Nutrition, Universidad de Navarra, 31008 Pamplona, Spain; malonso.17@alumni.unav.es (M.A.-J.); acerain@unav.es (A.L.d.C.); 2 Department of Pharmaceutical Technology and Chemistry, Research Group MITOX, School of Pharmacy and Nutrition, Universidad de Navarra, 31008 Pamplona, Spain; mfont@unav.es (M.F.); mgpenas@unav.es (E.G.-P.); 3 IdiSNA, Navarra Institute for Health Research, 31008 Pamplona, Spain

**Keywords:** mycotoxins, in silico, SOS/umu test, genotoxicity, prioritization

## Abstract

Humans are widely exposed to a great variety of mycotoxins and their mixtures. Therefore, it is important to design strategies that allow prioritizing mycotoxins based on their toxic potential in a time and cost-effective manner. A strategy combining in silico tools (Phase 1), including an expert knowledge-based (DEREK Nexus^®^_,_ Lhasa Limited, Leeds, UK) and a statistical-based platform (VEGA QSAR©, Mario Negri Institute, Milan, Italy), followed by the in vitro SOS/umu test (Phase 2), was applied to a set of 12 mycotoxins clustered according to their structure into three groups. Phase 1 allowed us to clearly classify group 1 (aflatoxin and sterigmatocystin) as mutagenic and group 3 (ochratoxin A, zearalenone and fumonisin B1) as non-mutagenic. For group 2 (trichothecenes), contradictory conclusions were obtained between the two in silico tools, being out of the applicability domain of many models. Phase 2 confirmed the results obtained in the previous phase for groups 1 and 3. It also provided extra information regarding the role of metabolic activation in aflatoxin B1 and sterigmatocystin mutagenicity. Regarding group 2, equivocal results were obtained in few experiments; however, the group was finally classified as non-mutagenic. The strategy used correlated with the published Ames tests, which detect point mutations. Few alerts for chromosome aberrations could be detected. The SOS/umu test appeared as a good screening test for mutagenicity that can be used in the absence and presence of metabolic activation and independently of Phase 1, although the in silico–in vitro combination gave more information for decision making.

## 1. Introduction

Mycotoxins (MTX) are low molecular-weight natural compounds produced as secondary metabolites by filamentous fungi, mainly *Aspergillus*, *Penicillium* and *Fusarium*. More than 300 compounds have been classified as mycotoxins; however, only a few have received attention as threats to human and animal health due to their toxic potential and worldwide contamination [1]. Indeed, at the EU level, only aflatoxins (AFs), ochratoxin A (OTA), patulin (PAT), deoxynivalenol (DON), zearalenone (ZEA), fumonisins (FUM), T-2 toxin (T-2) and HT-2 toxin (HT-2) are regulated in different foodstuffs [2]. Moreover, there is an increasing awareness of minimizing mycotoxin exposure [3]. The general population is widely exposed to mycotoxins, mainly through diet, as occurrence above a detectable level is considered to be up to 60–80% [4]. Furthermore, in the current context of climate change, mycotoxins might appear as an emerging risk [5], as new climate scenarios might modify the presence of regulated mycotoxins [6] but also might introduce emerging, and thus currently not regulated, forms [7]. In brief, there is a great diversity of mycotoxins that can contaminate food and feed, and therefore a variety of different toxicological effects to consider when prioritizing. For example, regarding DNA damage, some mycotoxins are known to be potent genotoxins (AFs) [8] and others seem to be clastogenic [9], while for others, their genotoxicity is unknown (such as, for example, 15-acetyldeoxynivalenol) [10]. Finally, humans are exposed to more than one mycotoxin. Several human biomonitoring studies conducted worldwide have demonstrated co-exposure to at least two mycotoxins; OTA + AFs being the most frequently analyzed mycotoxins in plasma (for a review, see [11]). Indeed, the combination of OTA + AFs together with AFs + FUM, DON + ZEA, and FUM + ZEA were the most observed combinations in cereals [12]. Data regarding their combined toxicity are still lacking or inconclusive, showing antagonist, additive or synergic effects depending on the tested species, cell model, or mixture [12,13].

Therefore, as the number of mycotoxins (including regulated and emerging/modified forms) and their possible combinations is very high, it is important to develop an efficient tiered strategy to prioritize the mycotoxins and their mixtures that should be first evaluated from a toxicological point of view [5]. Prioritization in a time- and cost-effective manner is pivotal for health-risk assessment of emerging risks.

Mycotoxins have numerous deleterious effects, including nephrotoxicity [14], hepatotoxicity [15], reprotoxicity [16], endocrine disruption [9], immunotoxicity [17], and intestinal toxicity [18]. The amount of resources and time necessary to characterize the toxicity could be reduced with prioritization strategies. One of the main health concerns for the general population is their toxicity at low concentrations and after long-term exposures [1]. In this regard, genotoxicity and carcinogenicity (Table 1) have been considered as important toxicological endpoints for mycotoxins, mainly due to the fact that aflatoxin B1 (AFB1), the first discovered mycotoxin, is considered to be one of the most potent human hepatocarcinogens known to date [19].

**Figure 1:**
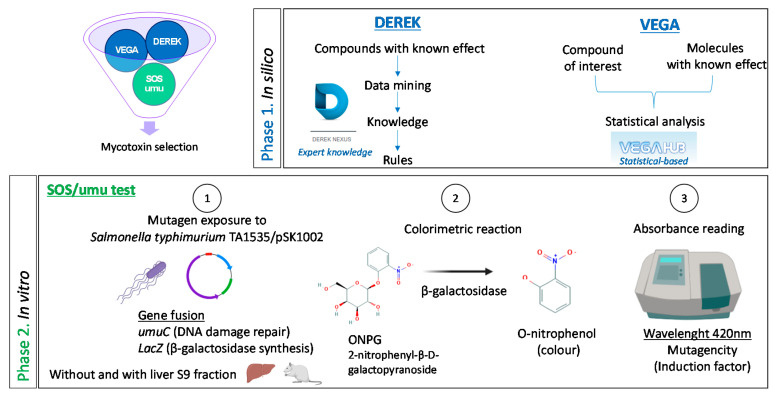
Strategy followed to prioritize mycotoxins based on their genotoxic potential. Phase 1 includes two different in silico tools: (i) DEREK Nexus^®^ Lhasa Limited, Leeds, UK (commercial) that uses rule-based predictions based on expert-knowledge, and (ii) the VEGA QSAR© Mario Negri Institute, Milan, Italy (free *online* platform) offering different models for statistical-based predictions. Phase 2 corresponds to the medium-throughput in vitro genotoxicity assay, the SOS/umu assay, based on a colorimetric reaction.

The aim of the present paper is to present and validate a strategy to prioritize mycotoxins based on their genotoxic potential. For that purpose, two in silico tools (Phase 1) and one medium-throughput in vitro genotoxicity test (Phase 2) were used (Figure 1).

Phase 1 was designed following ICH (International Conference on Harmonization of Technical Requirements for Pharmaceuticals for Human Use) M7 guideline for mutagenic assessment of impurities derived from drug development, which recommends the use of two complementary in silico methodologies: (i) expert rule-based and (ii) statistical-based [57]. Thus, Phase 1 was performed using the predictive tool DEREK Nexus^®^, a knowledge-based expert system for qualitative toxicity prediction, and VEGA QSAR©, a platform that offers different quantitative structure–activity relationship (QSAR) models. The objective of Phase 2 was to validate the results obtained in Phase 1 with an in vitro genotoxicity test. Normally, for genotoxicity testing of chemicals, a standard in vitro–in vivo battery is recommended (ICH S2R1 guideline) [58,59]. This battery always includes the Ames test (TG OECD 471) [60] as a first mutagenicity assay for detecting gene mutations, followed by other in vitro tests for detecting chromosomal aberrations, such as the micronucleus test (TG OECD 487) [61]. Depending on the outcome of the in vitro tests, follow-up in vivo assays might be needed. The complete recommended strategy is time-consuming and needs large amounts of test item (e.g., around 1 g only for the Ames test). The amount of test item is indeed a bottleneck in the field of mycotoxins, as some new emerging/modified forms are not commercially available and are only synthesized in small amounts by some research groups. Additionally, the commercially available mycotoxins are expensive, and very often the standardized tests following Organisation for Economic Co-operation and Development (OECD) guidelines have not been carried out in their totality (e.g., not using the five recommended bacteria strains in the Ames test). Therefore, for Phase 2, the SOS/umu assay was selected. It is a medium-throughput assay for genotoxicity screening [62,63], which allows us to evaluate the DNA damaging effect of several compounds (six per 96-well plate). It needs relatively small amounts of test item (around 2 mg), and results are available within two days. The test is carried out in *Salmonella typhimurium* TA1535/pSK1002 with and without metabolic activation (S9 mix). *Salmonella typhimurium* TA1535 incorporates a pSK1002 plasmid containing the umuC gene fused to a lacZ reporter gene. The umuC gene is activated as part of the bacterial SOS response. In turn, this promotes the β-galactosidase activity associated with lacZ, which is assessed by a colorimetric reaction. The test was selected because a high degree of agreement with the standardized Ames test (TG OECD 471) has been found [64].

The strategy has been applied to 12 mycotoxins, including the well-characterized aflatoxin B1 (AFB1) and ochratoxin (OTA), an emerging mycotoxin (sterigmatocystin-STER) with some genotoxicity red flags in the literature, and other regulated mycotoxins such as deoxynivalenol (DON), zearalenone (ZEA), fumonisin B1 (FB1), T-2 and HT-2 toxins. Moreover, some modified forms or structurally related metabolites have also been included: 3-acetyldeoxynivalenol (3ADON), 15-acetyldeoxynivalenol (15ADON), nivalenol (NIV) and fusarenon-X (F-X). For the analysis and reporting, mycotoxins were clustered based on their chemical structure.

## 2. Results

**Figure 2:**
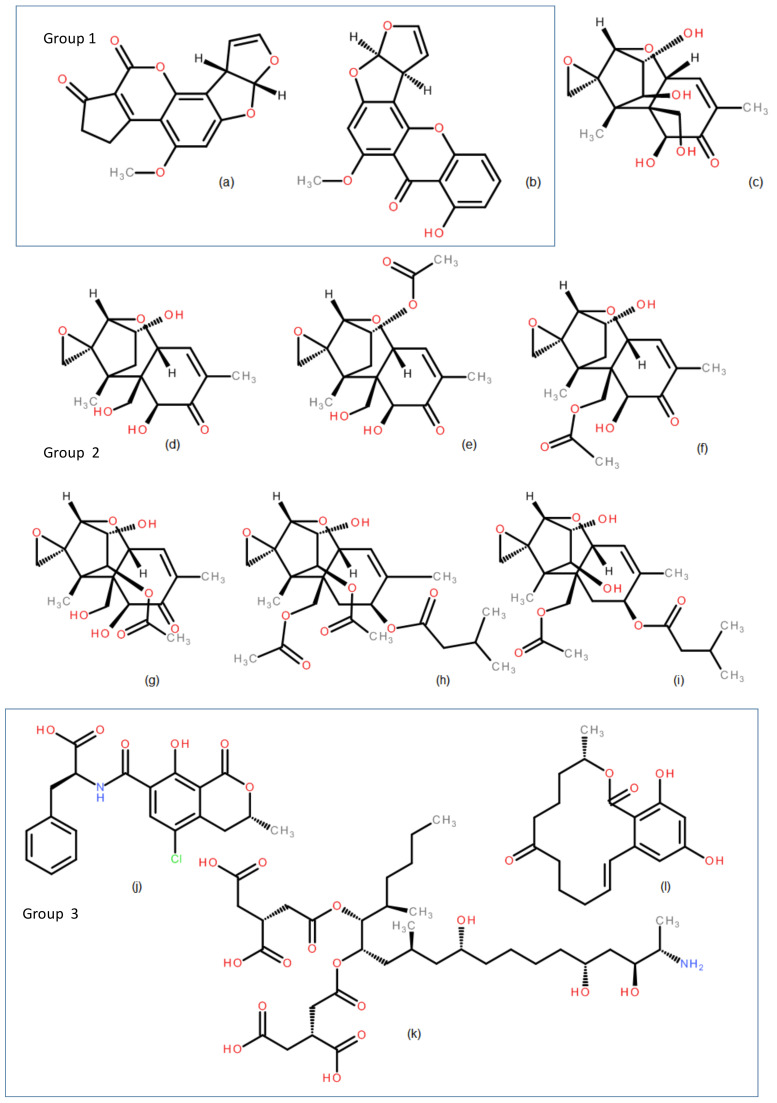
Structure for analyzed mycotoxins. Group 1: (**a**) AFB1; (**b**) STER; Group 2 (trichothecenes): (**c**) NIV; (**d**) DON; (**e**) 3ADON; (**f**) 15ADON; (**g**) F-X; (**h**) T-2; (**i**) HT-2 and Group 3: (**j**) OTA; (**k**) FB1; (**l**) ZEA.

The genotoxic potential was characterized in two phases, in silico (Phase 1) and in vitro (Phase 2). Before Phase 1, a structural activity relationship (SAR) analysis focused on Absorption, Distribution, Metabolism, Excretion and Toxicity (ADMET) descriptors was carried out for all the mycotoxins (for details, see Supplementary Data, Section 1). In Phase 1, all the mycotoxins were analyzed with DEREK Nexus^®^ and VEGA QSAR©. The in vitro assessment (Phase 2) was carried out with a SOS/umu test. For data reporting, the mycotoxins were clustered into three different groups based on their chemical structure (Figure 2).

### 2.1. Structural Analysis

From a structural point of view, the mycotoxins hereby studied (Figure 2) can be grouped into three different groups.

#### 2.1.1. Group 1. Furo[2 ,3-b]benzofuran derivatives, AFB1 and STER

**Figure 3:**
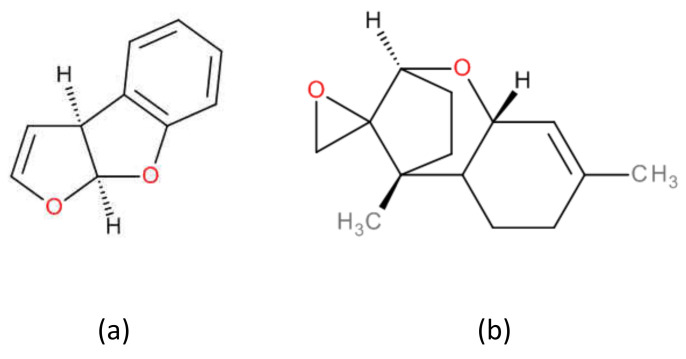
Structural scaffolds (**a**) for **Group 1:** furo[2,3-b]benzofuran (**b**) for **Group 2**: 1,5-dimethylspiro[8-oxatricyclo[7.2.1.0^2,7^]dodec-5-ene-12,2’-oxirane].

**Figure 4:**
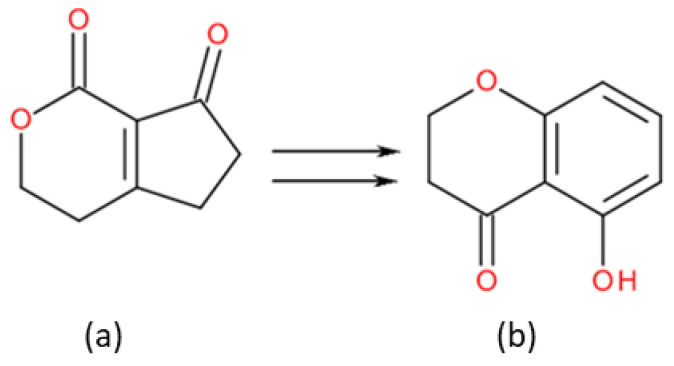
Structural variation (**a**) 3,4,5,6-tetrahydrocyclopenta[c]pyran-1,7-dione, AFB1 (**b**) 5-hydroxychroman-4-one, STER (see text for details).

AFB1 (Figure 2a) is an aflatoxin with a tetrahydrocyclopenta[c]furo[3’,2’:4,5]furo[2,3-h]chromene skeleton with oxygen functionality at positions 1, 4 and 11, whereas STER (Figure 2b) is an heteropentacyclic derivative whose skeleton comprises a xanthene ring system ortho-fused to a dihydrofuranofuran moiety; in both structures the central scaffold furo[2,3-b]benzofuran can be recognized (Figure 3a and Figure 4).

#### 2.1.2. Group 2. 1,5-Dimethylspiro[8-oxatricyclo[7.2.1.02,7]dodec-5-ene-12,2’-oxirane] derivatives: NIV, DON, 3ADON, 15ADON, F-X, T-2 and HT-2 (trichothecenes)

Taking as reference the structure of NIV (Figure 2c), (1S,2R,3S,7R,9R,10R,11S,12S)-3,10,11-trihydroxy-2-(hydroxymethyl)-1,5-dimethylspiro[8-oxatricyclo[7.2.1.02,7]dodec-5-ene-12,2’-oxirane]-4-one, removing the OH group in position 11 leads to DON (Figure 2d). The acylation of OH in position 3 of DON leads to 3ADON (Figure 2e), whereas the acylation of OH located at 15 position leads to 15ADON (Figure 2f). The acylation of OH located in position 3 of NIV leads to F-X (Figure 2g). The structural modification of the 2-hydroxymethyl group of NIV to 2-acetyloxymethyl, together with the acetylation of the OH moiety located at 11 and the modification of 1,5-dimethylspiro[8-oxatricyclo[7.2.1.02,7]dodec-5-ene-12,2’-oxirane]-4-one (Figure 3b) moiety to 1,5-dimethylspiro[8-oxa tricyclo[7.2.1.02,7]dodec-5-ene-12,2’-oxirane]-4-yl] 3-methylbutanoate moiety leads to T-2 toxin (Figure 2h). The hydrolysis of acyl moiety located at position 3 leads to HT-2 (Figure 2i).

#### 2.1.3. Group 3. Structurally Unrelated Toxins, OTA, FB1 and ZEA

OTA (Figure 2j) is a derivative resulting from the formal condensation of the amino group of L-phenylalanine with the carboxy group of (3R)-5-chloro-8-hydroxy-3-methyl-1-oxo-3,4-dihydro-1H-2-benzopyran-7-carboxylic acid.

FB1 (Figure 2k) is a diester derived from the condensation of the 1-carboxy groups of two molecules of propane-1,2,3-tricarboxylic acid with hydroxyl groups located at positions 14 and 15 of (2S,3S,5R,10R,12S,14S,15R,16R)-2-amino-12,16-dimethylicosane-3,5,10,14,15-pentol.

ZEA (Figure 2l) is a macrolide comprising a fourteen-membered lactone fused to 1,3-dihydroxybenzene.

### 2.2. In Silico Toxicology (Phase 1)

Table 2 and Table 3 show detailed results obtained with DEREK Nexus^®^ and VEGA QSAR©, respectively.

#### 2.2.1. Group 1 (AFB1 and STER)

AFB1 and STER had similar pattern predictions with DEREK Nexus^®^, related to their structural similitudes. In fact, the presence of a bisfuranoid moiety (Alert 201) and an alkyl–aldehyde precursor (Alert 306) can be considered as a potential toxophore. Therefore, plausible mutagenicity in vitro (in both bacteria and mammalian cell models) and chromosomal aberrations in vitro were retrieved. The non-specific genotoxicity in vitro was also considered as plausible, while no predictions were obtained for chromosome damage in vivo for both mycotoxins (Table 2).

AFB1 also has an α, β-unsaturated ketone moiety that implies a specific alert (309), a potential toxophore, which reinforces positive genotoxic potential for this toxin.

Regarding VEGA QSAR© outcomes, all experimental outputs agreed on AFB1 mutagenicity, for which information about its experimental mutagenicity was detected (see Table 3). All predictions showed positive outputs with good reliability (See Table 4).

According to the SarPy/IRFMN approach, AFB1 shows a 2-vinyloxyethylbenzene fragment which implies a structural alert (SM35) for mutagenicity. Moreover, some non-mutagenicity alerts are detected: SM141, SM154, SM158, SM177, SM187 and SM196 (see Table S3 for details).

According to the ISS approach, this compound shows a coumarin moiety (SA30) related to mutagenicity (Table S4).

With respect to STER, the experimental outputs from three models (CAESAR, SarPy/IRFMN and ISS) agreed on its mutagenic potential. SarPy/IRFFMN detected, in addition to the SM35 alert that is common with AFB1, the presence of (2-hydroxyphenyl)-phenyl-methanone (SM59) and 9H-xanthen-3-ol (SA84) moieties, which implies mutagenicity. Meanwhile ISS detected the presence of a fragment related to the structure of demonstrated DNA intercalating agents (SA59, xanthones, thioxanthones, acridones). The prediction outputs from these models were also positive with good reliability (Table 4).

Based on the abovementioned results, the consensus obtained with VEGA QSAR© was mutagenicity for both mycotoxins (Table 4).

Therefore, regarding Phase I, the overall classification for AFB1 and STER could be “mutagenic” based on outputs from both in silico tools.

#### 2.2.2. Group 2 (Trichothecenes: NIV, DON, 3ADON, 15ADON, F-X, T-2 and HT-2 Toxins)

The prediction from DEREK Nexus^®^ for these compounds detects the presence of the epoxide (Alert 019) that decorates the common scaffold. Therefore, a plausible mutagenicity and chromosomal damage was predicted in vitro and in vivo.

In addition, NIV, DON, 3DON, 15ADON and F-X showed an α, β-unsaturated ketone (Alert 309) toxophore, also present in AFB1, which reinforces the plausibility of a genotoxic potential in vitro and in vivo (see Table 2). Therefore, there was a positive genotoxic potential of the mycotoxins from this group, after screening phase 1 with DEREK Nexus^®^.

Considering VEGA QSAR© outcomes, the CAESAR approach detected the presence of the epoxide (SA7) (Table S2) in all compounds belonging to this group, and therefore labelled them as suspects of mutagenicity. With respect to the SarPy/IRFMN model, it detected two common SM alerts, related to the presence of oxiran-2-ylmethanol (SA92) and epoxide (SA97) moieties. These alerts are accompanied by a series of non-mutagenicity ones (see Table 3 and Table S3). The ISS approach also detected epoxide (SA7) in all the analyzed structures, as well as alpha, beta unsaturated carbonyls (SA10), except for T-2 and HT-2 (Table S4).

According to the aforementioned data, some structural alerts have been detected for this group of mycotoxins and the predictions labelled the mycotoxins as “mutagenic” in 3 of the 4 models used (CAESAR, SarPy/IRFMN and ISS). Unfortunately, the quality obtained for these predictions (Table 4) was low, as all the mycotoxins were “out” or “almost out” of the applicability domains (AD) of the aforementioned models, with applicability domain indexes (ADI) of 0 in many cases. The AD of a model is the physico-chemical, structural or biological space, knowledge or information on which the training set of the model has been developed, and for which it is applicable to make predictions for new compounds. It is a calculated region of the chemical space of the training dataset used to make the model [65]. Moreover, there was no concordance between the prediction and experimental outputs retrieved for NIV, DON and F-X. For instance, NIV and DON showed no mutagenicity from experimental outputs of three models (CAESAR, SarPy/IRFMN and KNN/Read-across while F-X obtained negative experimental outputs from two models (CAESAR and SarPy/IRFMN). The highest reliability prediction (ADI = 1) was provided by KNN/Racross model for NIV, DON, 3ADON, 15ADON and F-X. The prediction of this model matched with the experimental outputs as well with the final consensus outcome from VEGA QSAR©: non- mutagenic (see Table 4).

Regarding T-2 and HT-2, the predictions were also considered to be of low quality, as both mycotoxins were “out” or “almost out” in the AD of the four models. Moreover, T-2 experimental outputs from two models (CAESAR and SarPy/IRFMN) indicated an absence of mutagenicity, whereas HT-2 did not have experimental outputs in any model. Despite having similar prediction patterns, the absence of experimental outputs for the HT-2 toxin induced contradictory conclusions in the consensus outcomes from VEGA QSAR©: the T-2 toxin was considered not mutagenic, while HT-2 was labelled mutagenic.

Overall, for this group of mycotoxins, DEREK^®^ labelled them as mutagenic while the consensus analysis retrieved from VEGA QSAR© labelled them as non-mutagenic, except in the case of HT-2. Therefore, considering Phase I the overall classification for this group of mycotoxins, could be “equivocal” based on the outputs obtained in both in silico tools.

#### 2.2.3. Group 3 (OTA, FB1 and ZEA)

The overlap predictions among OTA, ZEA and FB1 from screening phase 1, agreed on the absence of structural alert activation in any genotoxicity endpoint. With DEREK Nexus^®^, there was just a specific label of no effect for in vitro mutagenicity in bacteria (Table 2). Therefore, the conclusion in this group is a negative genotoxicity potential in all cases.

In relation to the outcomes obtained with VEGA QSAR© for OTA, ZEA and FB1, two models (ISS and KNN/Read-across) gave a sufficiently reliable (ADI = 1) negative prediction (Table 4). The experimental outputs showed non-mutagenic activity in the same two models for OTA and FB1. In the case of ZEA, all experimental outputs from the four models agreed on negative mutagenicity. Indeed, the consensus retrieved from VEGA QSAR© was absence of mutagenicity for the three mycotoxins (Table 4).

Therefore, the overall classification from Phase 1 for OTA, ZEA and FB1 was “non- mutagenic”.

### 2.3. In Vitro Genotoxicity Screening (Phase 2): SOS/umu Test

#### 2.3.1. Exposure Concentrations and Solubility

The maximal concentration to be tested in the SOS/umu test is 1000 µg/mL per well. To this aim, stock solutions at 40 mg/mL in dimethyl sulfoxide (DMSO) (water for FB1) were prepared except for AFB1 (5 mg/mL), STER (5.88 mg/mL), DON (35 mg/mL) and ZEA (37 mg/mL), which were insoluble at higher concentrations.

In the case of NIV, 3ADON, 15ADON and the HT-2 toxin, as they were highly soluble and non-toxic for bacteria at the maximal concentration of 40 mg/mL, two higher concentrations of 160 and 80 mg/mL (corresponding to 4000 and 2000 µg/mL, respectively, in the well-plate) were also included in an independent experiment.

Some mycotoxins precipitated during the SOS/umu test. The concentrations containing precipitates were not considered for analysis.

#### 2.3.2. SOS/Umu Test

**Figure 5:**
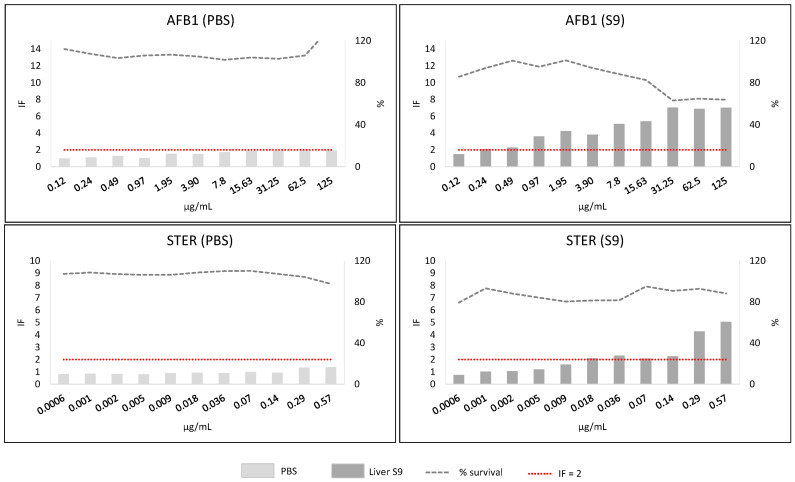
Results from SOS/umu test with (dark grey) or without S9 (light grey) activation for AFB1 and STER. Bacterial survival is shown as percentage (%). Concentrations are considered non-toxic if survival is >80%. A compound is considered genotoxic if the induction factor (IF) is ≥2 at non-toxic concentrations for the bacteria in any of the conditions tested.

**Figure 6:**
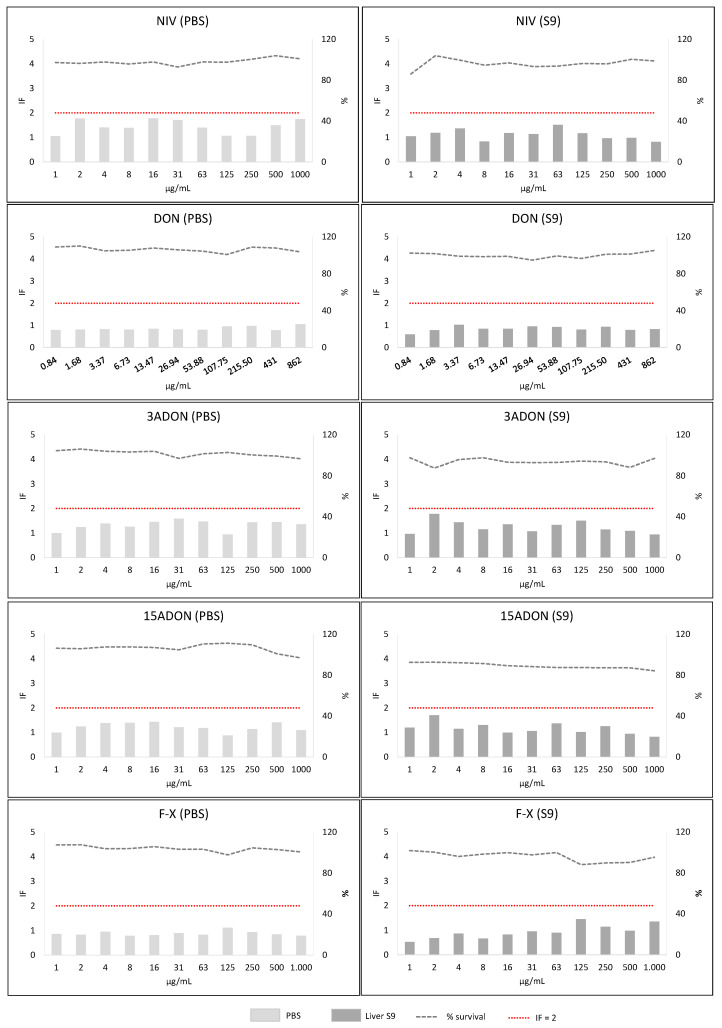
Results of SOS/umu test with (dark grey) or without S9 (light grey) activation for NIV, DON, 3ADON, 15ADON and F-X. Bacterial survival is shown as percentage (%). Concentrations are considered non-toxic if survival is >80%. A compound is considered genotoxic if the induction factor (IF) is ≥2 at non-toxic concentrations for the bacteria in any of the conditions tested.

**Figure 7:**
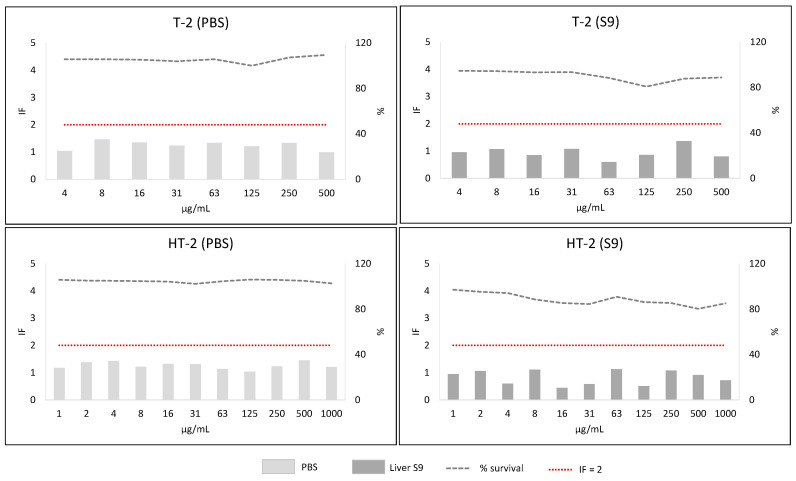
Results from SOS/umu test with (dark grey) or without S9 (light grey) activation for T-2 and HT-2. Bacterial survival is shown as percentage (%). Concentrations are considered non-toxic if survival is >80%. A compound is considered genotoxic if the induction factor (IF) is ≥2 at non-toxic concentrations for the bacteria in any of the conditions tested.

**Figure 8:**
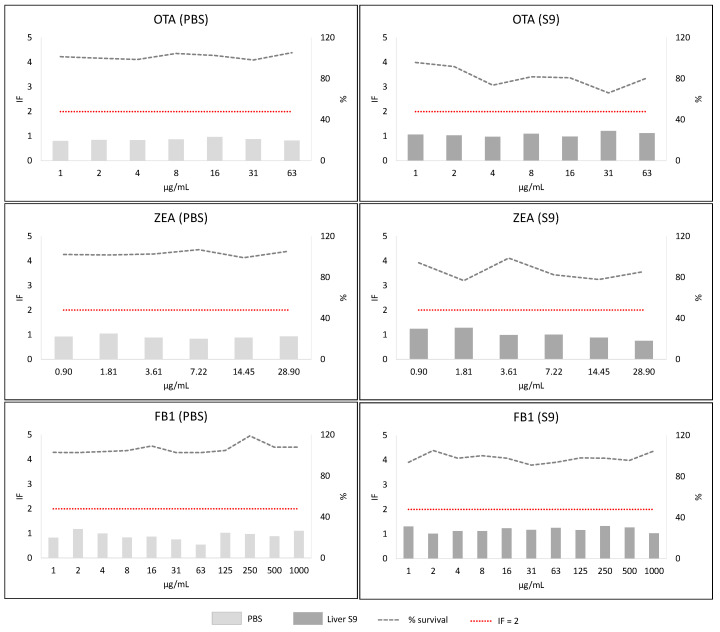
Results from SOS/umu test with (dark grey) or without S9 (light grey) activation for OTA, ZEA and FB1. Bacterial survival is shown as percentage (%). Concentrations are considered non-toxic if survival is >80%. A compound is considered genotoxic if the induction factor (IF) is ≥2 at non-toxic concentrations for the bacteria in any of the conditions tested.

Screening phase 2 corresponded to the SOS/umu test. From the eleven concentrations tested for each mycotoxin, the wells containing precipitates of the mycotoxin were not considered for analysis (and thus are not shown in Figure 5, Figure 6, Figure 7 and Figure 8). Mycotoxins were considered genotoxic if the induction factor (IF) was higher than 2 at non-toxic concentrations (bacterial survival higher than 80%) at any of the concentrations tested without or with metabolic activation. In all the experiments, all the controls used were correct (IF < 2 for negative and >2 for positive controls).

#### 2.3.3. Group 1 (AFB1 and STER)

AFB1 showed a positive genotoxic concentration response in both without (PBS) and with metabolic activation (S9), but with a much greater response with metabolic activation (positive from 0.24 µg/mL). The three highest concentrations tested (31.25–125 µg/mL) with the metabolic activation were toxic for the bacteria. STER precipitated at the highest concentrations (from 9.2 to 147 µg/mL) and was very toxic to the bacteria (from 0.57 µg/mL) with metabolic activation (data not shown), and thus lower concentrations were tested (Figure 5). STER was not genotoxic without metabolic activation but showed genotoxicity with metabolic activation (IF > 2 from 0.14 µg/mL).

#### 2.3.4. Group 2 (NIV, DON, 3ADON, 15ADON, F-X, T-2 and HT-2 Toxins)

All the mycotoxins showed IF lower than 2 in the absence (PBS) and presence of metabolic activation (S9). However, NIV, 3ADON, 15ADON, T-2 and HT-2 toxin showed some results IF ≥ 1.5. Taking into account these results, as well the Phase I outcome, they were considered as equivocal. Moreover, they were very soluble and non-toxic for bacteria. Therefore, a second experiment at higher concentrations (maximal concentration 4000 µg/mL) was carried out. The mycotoxin T-2 precipitated at 1000, 2000 and 4000 µg/mL. The rest of the mycotoxins did not show precipitates at any of the concentrations. This second experiment showed IF below 1.5 in all cases (Figure S1) and allowed us to consider these mycotoxins as “non-mutagenic”.

#### 2.3.5. Group 3 (OTA, FB1 and ZEA)

OTA, ZEA and FB1 showed a clear negative genotoxic response in the absence (PBS) or presence of metabolic activation (S9). In all cases IF was well below 2.

Overall, taking into account the results from the SOS/umu test, AFB1 and STER could be considered clearly genotoxic, while DON, F-X, OTA, ZEA and FB1 were clearly non-genotoxic. Some equivocal results were obtained with NIV, 3-ADON, 15-ADON and T-2/HT-2 toxins but were confirmed as negative in a second experiment.

## 3. Discussion

The identification and initial toxicological characterization of emerging risks is at the heart of public health protection [66]. The current climate change scenario might also affect human exposure to mycotoxins as it is known that changes in temperature, humidity, rainfall and carbon dioxide production impact on fungal behavior and consequently on mycotoxin production. Moreover, it is known that animals and humans are not only exposed to one single mycotoxin, but to cocktails of different contaminants. On the other hand, the current paradigm shift from traditional toxicology testing (in vivo) towards alternative methods following the 3R principles (replacement, reduction and refinement of animal testing) brings forth the use of in silico and in vitro methods. The current genotoxicity testing battery used for pharmaceuticals [58] and for food safety assessment [59] is a stepwise battery that begins with in vitro tests, generally an Ames test [60] and micronucleus tests [61], followed by an in vivo test if needed. The genotoxic endpoint assessed in this battery are mainly point mutations (Ames test) and chromosome aberrations (micronucleus assay). However, the in vitro part of this battery might be time-consuming (from 2–3 weeks per complete assay) and high amounts of test items are generally needed. The substance availability is indeed a usual problem for mycotoxin evaluation as many of them are very expensive or not commercially available. Thus, there is a need to develop tiered approaches for prioritizing mycotoxins or mixtures that need to be further studied.

It is known that in silico methods are not yet ready for fully replacing in vitro and in vivo testing. For this reason, the ICH M7 [57] strategy reccomends the use of two in silico methods (expert knowledge-based and statistical-based) for genotoxicity prediction of pharmaceutical impurities. Morever, it has also been demonstrated that the combination of methods increases the performance of the models used for genotoxicity prediction of food contact material, although this was more relevant for QSAR methods [67].

Therefore, two different already available in silico tools were used in the present study.

DEREK Nexus^®^ is commercial, based on expert knowledge and it is normally used by pharmaceutical companies as a part of an ICH M7 workflow to detect mutagenic impurities. In our case, only OTA, ZEA and FB1 were considered inactive for mutagenicity in vitro, with no prediction for the rest of genotoxiticity endpoints. The rest of the mycotoxins showed structural alerts for mutagenicity and chromosome damage, however with a lower level of evidence within the positive range (that goes from plausible to certain), as they were labeled as “plausible”. Therefore, DEREK allowed us to clearly group the mycotoxins into two clusters: negative for gene mutation (OTA, ZEA and FB1) or mycotoxins with some alerts for genotoxicity.

VEGA QSAR© is free, statistical-based and includes several models for mutagenicity prediction (CAESAR, SarPy/IRFMN, ISS, KNN/Racross), as well as an independent algorithm to assess the reliability of the model predictions through the ADI. The ADI algorithm works on all the separate models and shows similar compounds, assesses the statistical-based results on the similar compounds, and analyses some relevant chemical features in the target compound and its related compounds. Thereafter, an automatic evaluation for read-across is completed, and thus this tool can be used for read-across, independently from the prediction obtained through the models. Moreover, VEGA consensus was also used as previous studies have demonstrated that when the performance varies among the different mutagenicity models used, it increased the prediction performance. The weighted consensus read-accross result is obtained by taking into account the result of each of the models and their associated compound-specific ADI [67]. In our case, very good predictions were obtained for AFB1 (mutagenic) and ZEA (non-mutagenic), with ADI of 1 in all the models used. Some of the other mycotoxins were also predicted with good quality (ADI = 1) in some of the models, such as STER (mutagenic) in CAESAR, SarPy/IRFMN and ISS; NIV and DON (both labelled as non-mutagenic) in KNN/read-across; and OTA and FB1 (non-mutagenic) in ISS and KNN/read-across. In the case of STER, OTA and FB1, the rest of the models gave moderate predictions (ADI between 0.72 and 0.82), but in all cases, the prediction matched the results obtained with the models showing higher ADI, as well the experimental data included in the different databases. Moreover, ADI levels higher than 0.75 have been considered to be of enough quality for genotoxicity prediction [67]. Thus, it can be considered that there is enough evidence to classify STER as mutagenic, while OTA and FB1 can be labelled as non-mutagenic. However, the level of evidence to classify the rest of mycotoxins was much lower. Indeed all the group 2 of structurally related mycotoxins (trichothecenes: NIV, DON, 3ADON, 15ADON,l F-X, T-2 and HT-2) were totally out of the domain of two of the models (CAESAR and SarPy/IRFMN) that indeed classified these molecules as mutagenic or suspected to be mutagenic. ISS, a model that showed a low or moderate quality prediction (ADI below 0.75 for NIV, DON, 3ADON and 15ADON, and ADI higher than 0.75 for F-X, T-2 and HT-2), and also classified them as mutagenic. The model showing higher levels of quality predictions for this group of mycotoxins was KNN/Racross with ADI between 0.78 and 1; however, it classified them as non-mutagenic. The KNN/Racross classification was in concordance with the experimental data retrieved for these mycotoxins in the databases of some of the models, except for 3ADON, 15ADON and HT-2 for which experimental data were lacking. Thus, based on the results obtained with the in silico tools, we considered these mycotoxins as equivocal for mutagenicity.

One important aspect to point out is that in silico tools for mutagenicity prediction are mainly built based on in vitro experimental data, mainly from the Ames test [68,69] [70,71,72], and thus this should be taken into account when interpreting the classification “mutagenic” or “ non-mutagenic” as the prediction is mainly focused on point mutation, but not for other genotoxic endpoints.

Therefore, in order to validate the in silico Phase 1, the SOS/umu test, an in vitro test with a high concordance with the Ames test was used [64]. Indeed, a comparison of this assay with other SOS Chromotests and the Ames test itself revealed that the sensitivity of the SOS/umu test was close to those of the Ames test strain TA98, both with and without metabolic activation. Moreover, the authors considered that this assay was especially suitable for screening complex environmental samples as it demonstrated a significant reduction in material expenses and workload compared to the Ames test [73].

The results obtained with the SOS/umu test confirmed the mutagenicity of AFB1 and STER (Group 1 of mycotoxins). This test added some extra information not obtained in Phase 1, regarding the need for metabolic activation, as both mycotoxins were only mutagenic when the S9 mix was used. The assay also confirmed the lack of mutagenicity of mycotoxins from Group 3 (OTA, ZEA and FB1), as the IF were always below the threshold for genotoxicity (IF < 2). Indeed, the IF were always lower than 1.5 at all the concentrations tested with and without metabolic activation. Regarding Group 2, IF < 2 were obtained for all the mycotoxins. However, values around or higher than 1.5 were reached. Therefore, a second experiment with higher concentrations was carried out with the mycotoxins that were soluble and non-toxic to the bacteria (NIV, 3ADON, 15ADON and T-2/HT-2 toxins). They were confirmed as “non-mutagenic”.

The classification of the mycotoxins following the proposed strategy is in agreement with the published literature as well, with most risk assessments carried out by different international organizations.

The well-known AFB1, produced by the *Aspergillus* species, has been consistently found to be genotoxic and is considered a human carcinogen via DNA adduct formation after bioactivation [8,74,75]. STER, which is a precursor/intermediate of the aflatoxin (acetate-malonate) synthesis pathway, contaminates food and feed commodities but has not been fully characterized. Some studies confirmed the STER genotoxic potential, but the mode of action has not been clearly elucidated [76,77,78]. Both mycotoxins have been consistently found to be positive with Ames and SOS tests (see Table 1).

Another mycotoxin produced by the *Aspergillus* species is ochratoxin A (OTA). It is considered a potent nephrocarcinogen in rodents [14], although its mode of action is still unknown and a matter of debate [79]. The majority of the Ames and SOS tests carried out to date are negative, except for one study in which mice kidney microsomal fraction was used as metabolic activation [50]. In one SOS Chromotest, [33] OTA showed a slight increase in SOS response but with no dose effect.

The other mycotoxins selected to be evaluated with the proposed strategy are produced by *Fusarium:* zearalenone (ZEA), fumonisins and trichothecenes (types A and B and modified forms). Trichothecenes are structurally related toxins divided in four classes, with different chemical structures, biological effects and toxigenic fungi [80]. Types A and B have in common that they have an oxygen function at C-3 and are produced by the same biosynthetic route [81]. T-2 and HT-2 are type A trichothecenes. T-2 has been assessed through Ames and SOS tests, and in both cases seems to be negative. However, no data for HT-2 have been found (see Table 1). These findings are in line with our negative SOS/umu results and with the most recent European Food Safety Authority (EFSA) evaluation in which it is clearly stated that the T-2 toxin has been shown to be negative in bacterial mutation assays [82]. However, it should be noted that several studies highlighted that T-2 induces chromosomal damage in vitro and in vivo. However, it is not clear yet if the observed effects are a consequence of interaction of T-2 toxin with genetic material or are secondary to inhibition of protein synthesis or cytotoxicity [82]. Our in silico results have highlighted structural alerts related to epoxides, structures known to interact with DNA, in both in silico tools. Indeed, DEREK predicted plausible chromosome damage in vitro and in vivo for both mycotoxins. For VEGA QSAR©, it should be noted that the predictions were not of high enough quality.

Regarding the genotoxic potential of type B trichothecenes, the published literature indicates that it is inconclusive or unknown [10,83]. Our bibliographic search retrieved information indicating that DON, 3ADON and F-X were negative when tested with Ames and/or SOS tests. No data of the mutagenic potential of NIV and 15ADON has been retrieved from our systematic search (see Table 1). In the last EFSA evaluation [10], the panel concluded in agreement with our results from the SOS/umu test that DON is not mutagenic in bacteria or mammalian cells but considered that it causes chromosomal effects and DNA damage assessed by the comet assay in vitro, and that the effect was related to oxidative stress. Several in vivo studies carried out with DON gave inconclusive results [10]. Regarding the acetylated forms, very few studies have been carried out; however, they point to negative mutagenic effects in bacteria but again, some chromosomal aberrations have been observed in vitro for 3ADON. No in vivo data were available for the acetylated forms [10]. In the case of NIV, negative results have been found in assays detecting point mutations [84]. Some positive genotoxic results have been observed in other assays, in vitro or in vivo, but the most recent evaluation from EFSA considered that the overall weight of evidence is that nivalenol is unlikely to be genotoxic [84]. Very few data points regarding F-X have been found. The only Ames test carried out (Table 1) agrees with our SOS/umu results.

Finally, regarding ZEA, it is considered a xenoestrogen, due to structural similarities with these natural components [85]. Again, ZEA showed negative results in bacterial gene mutation assays; however, it has been considered as a clastogenic compound [9]. Our strategy allowed us to detect a negative response for bacterial mutagenicity with DEREK and SOS/umu and was classified as non-mutagenic in the consensus from VEGA. However, there were no predictions for chromosomal damage in DEREK or lack of mutagenicity alerts in VEGA.

Among fumonisins, fumonisin B1 (FB1) is the most prevalent and most toxic derivative [86]. This mycotoxin is also considered non-mutagenic in bacterial assays (Table 1) [87], and thus our strategy was able to correctly classify it as non-mutagenic. However, as in the case of ZEA, we were not able to predict or to highlight alerts regarding other type of genotoxic endpoints; FB1 gave positive results for chromosomal damage and DNA breaks [87].

## 4. Conclusions

A strategy combining two in silico tools, i) the commercial tool DEREK Nexus^®^ and ii) the publicly available VEGA QSAR©, followed by the in vitro SOS/umu test, allowed us to classify in a time- and cost-effective manner 12 mycotoxins based on their mutagenic potential (mutagenic, non-mutagenic, equivocal). The outputs correlated very well with the results obtained in the literature for the Ames test, which detects point mutations. The strategy does not allow the classification of mycotoxins based on their genotoxicity at the chromosome level; however, some alerts related to this endpoint could be also identified. To this aim, more data need to be included in the in silico models and a medium/high throughput assay for chromosome aberrations is needed.

Phase 1 (in silico) of the strategy revealed that some well-studied mycotoxins (trichothecenes) are out of the domain of applicability of the models. Thus, it is important to carefully check if the proposed strategy, or at least Phase 1, might be valid for emerging or modified mycotoxins. This first phase also allowed us to identify experimental data gaps for some mycotoxins (trichothecenes).

Regarding Phase 2 (in vitro), the SOS/umu test appeared as a very good screening test for the detection of the mutagenic potential of mycotoxins, as the obtained results correlated with the published Ames test results and the relatively old studies carried out with SOS tests. This test can be used independently of Phase 1.

The combination of the in silico (Phase 1) and in vitro (Phase 2) methods allowed us to obtain more complete information for decision making in the prioritization process.

Overall, these kinds of strategies could be useful for the prioritization of emerging/modified mycotoxins and their mixtures, in order to select the ones that need to be first evaluated for genotoxicity.

## 5. Materials and Methods

### 5.1. Chemicals and Reagents

The chemical reagents and solvents used for the SOS/umu test were obtained from different commercial companies. DMSO and Na_2_CO_3_ were purchased from PanReac AppliChem (Barcelona, Spain). Bactotryptone for TGA medium was obtained from Bectone Dickinson (Madrid, Spain), dextrose and NaCl from PanReac AppliChem (Barcelona, Spain). Ampicillin, ONPG (2-nitrophenyl- β-D-galactopyranoside), the B-buffer ingredients (Na_2_HPO_4_ 2H_2_O, NaH_2_PO_4_ H_2_O, MgSO_4_ 7H_2_O, sodium dodecyl sulfate, β-mercaptoethanol) in which the ONPG was dissolved and the positive controls 2-aminoanthracene (2AA) and 4-nitroquinoline-N-oxide (4NQO) were purchased from Sigma-Aldrich (Darmstadt, Germany). The B-buffer also comprises KCl, which was purchased from PanReac AppliChem (Barcelona, Spain). In this assay, a liver fraction (S9 fraction) from Sprague Dawley male rats of 6–8 weeks of age induced with Arocolor 1254 was purchased from Trinova Biochem (Giessen, Germany). The S9 mix (8%) was prepared with phosphate buffer (NaH_2_PO_4_ H_2_O, Na_2_HPO_4_ 2H_2_O), glucose-6-phosphate and NADP solutions, of which compounds were obtained from Sigma-Aldrich (Darmstadt, Germany) and saline solution ingredients (MgCl_2_ 6H_2_O, KCl) from PanReac AppliChem (Barcelona, Spain). Afterwards, the S9 mix was filtered through a 0.45 µ filter. Genetically modified *Salmonella typhimurium* 1535/pSK1002 was purchased from the *German Collection for microorganisms and Cell cultures* (DSMZ 9274) (Berlin, Germany) and used as SOS/umu test strain. The bacteria contain a plasmid in which two genes are fused, one involved in DNA repair and the other accountable for β-galactosidase activity [88]. Therefore, SOS repair activity is monitored through β-galactosidase induction activity determined with spectrophotometric measures. All mycotoxins were purchased from Sigma Aldrich (Darmstadt, Germany) in powder dissolved in DMSO or water and maintained at −20 °C until use. The reference commercial number and CAS number is indicated in each case: OTA (O1877; CAS: 303-47-9), ZEA (Z2125; CAS: 17924-92-4), FB1 (F1147; CAS: 116355-83-0), DON (D0156; CAS: 51481-10-8), F-X (33438; CAS:23255-69-8), AFB1 (A6636; CAS: 1162-65-8), STER (S3255; CAS:10048-13-2), NIV (32929; CAS: 23282-20-4), 3ADON (A6166; CAS:50722-38-8), 15ADON (32928; CAS: 88337-96-6), T-2 toxin (33947; CAS: 21259-20-1), and HT-2 toxin (T4138; CAS: 26934-87-2). The solutions were prepared in a chemical cabinet [89]. During the procedure, the manipulator wore a protective mask filter face piece type 3 (FFP3) and double gloves.

### 5.2. In Silico Toxicology (Phase 1)

#### 5.2.1. DEREK Nexus^®^

The expert knowledge rule-based software employed was Derek Nexus^®^ (v 6.0.1, Lhasa Limited, Leeds, UK,) [90,91]. It is a commercial model based on the application of alerts and reasoning rules which covers alerts for a variety of toxicological endpoints. An alert consists of a toxicophore (a substructure known or thought to be responsible for the toxicity) and is associated with literature references, comments and examples from toxicological experts. The rules are based either on hypotheses relating to mechanisms of action of a chemical class or on observed empirical relationships. The final toxicity assessment is a result of a two-part process: (i) the program checks whether any alerts from the knowledge base appear in the query compounds, and (ii) the reasoning model is applied in order to determine the likelihood of the compound’s toxicity (expressed as the level of likelihood) [92]. In the present study, the endpoints assessed were chromosomal aberrations (in vitro; in vivo), mutagenicity (in vitro for bacterium and mammals; in vivo) and non-specific genotoxicity (in vitro; in vivo). Prediction results ranging from “plausible” to “certain” were considered as mutagenic, whereas results from “inactive” to “doubted” were considered as non-mutagenic. The prediction “equivocal” was kept as a separate group. In the case of inactive predictions (query structure does not match any structural alerts or examples in Derek which show activity in a bacterial reverse mutation assay), DEREK Nexus^®^ also provided information of misclassified or unclassified features. Misclassified refers to features in the molecule that are found in non-alerting mutagens in the Lhasa reference data, while unclassified refers to features that have not been found in the reference set. In the present study, the label “no prediction” was used for endpoints not firing any alerts at the selected reasoning level.

#### 5.2.2. VEGA QSAR©

The free VEGA QSAR^®^ platform (Version 1.1.5., Mario Negri Institute, Milan, Italy [93] was the second tool employed for Phase 1. For the current analysis, four available mutagenicity models were used: CAESAR 2.1.13; SarPy/IRFMN 1.0.7; ISS 1.0.2; KNN/Read-across 1.0.0. All models provided a qualitative class label for each mycotoxin evaluated: non-mutagenic, mutagenic and suspected to be mutagenic. These class labels were provided for both prediction and experimental outputs [93].

The level of prediction reliability was also estimated. For that purpose, the VEGA independent algorithm (Consensus 1.0.2 based on read across) was used. This algorithm works on all the separate models. Concretely, it assesses three models’ pillars related to: (i) the property (mutagenicity) to be studied (concordance, accuracy and structural alerts), (ii) chemical information (similarity) and (iii) function linking the property and the chemical (algorithm). The resulting parameters of the elements assessed are merged into the applicability of domain (AD) and are classified as into, out and could out of domain. In order to evaluate the AD of the models, two methods were also gathered by VEGA: (i) the descriptors range check (DRC) that evaluates if the descriptors of the target compound has values in the range of those related to the substances in the training set (TS), and (ii) atom-centred fragments (ACF) an index that takes into account the presence of one or more fragments that are not found in the model TS, or that are rare fragments. The applicability of domain index (ADI) could be expressed in a quantitative (numerical) value from 0 to 1—equal to a compound in the training set of the model—of each model for a particular compound [93]. In order to characterize both indexes, consider the profile of fundamental physicochemical properties of the training set compounds as well as their structural features and mechanistic disposition [94] In the present study, the AD and ADI values were first evaluated. If ADI > 0.8 and AD was into the applicability of domain, the prediction was considered to be strongly supported [93]. If both conditions were not fulfilled, the other reliability parameters were assessed: similarity (Sim) with the two/three most similar compounds in the training set (TS), accuracy (Acc) in the prediction for the two/three most similar compounds in TS and concordance (Cc) between the predicted value and the experimental values of the three or two (depends on different models) most similar compounds in the model TS.

Results are expressed in a quantitative way (0 to 1) for ADI and qualitative way for AD (into, out, could out) and for DRC (true, false). Reliability of the prediction, based on the overall results obtained, is expressed as good, low and moderate.

Special emphasis was given to the concordance of the experimental output retrieved from each model and the prediction. Additionally, the presence of structural alerts (SA) provided by the ISS model and mutagenicity relevant substructures (SM) provided by SarPy/IRFMN model were considered.

### 5.3. In Vitro Genotoxicity Screening (Phase 2)

#### 5.3.1. Exposure Concentrations and Solubility Test

All stock solutions were prepared in DMSO, except FB1 which was dissolved in water. The selected maximum concentration to be tested in the SOS/umu assay was 40 mg/mL (corresponding to 1000 µg/mL in the 96-well plate). For insoluble substances, the highest soluble concentration was used. Insolubility was assessed as precipitation (or turbidity) in the solvent and evident to the unaided eye over a dark background. For very soluble mycotoxins (NIV, 3ADON, 15ADON, T-2, HT-2) that were not toxic for bacteria and induced an equivocal response, two extra concentrations were also tested: 160 and 80 mg/mL (corresponding to 4000 and 2000 µg/mL in the 96-well plate, respectively). The positive control stock solutions were prepared in DMSO: at 500 µg/mL (12.5 µg/mL in 96- well plate) for 2-aminoanthracene (2-AA) (Sigma-Aldrich, Germany) and at 100 µg/mL (2.5 µg/mL) for 4-nitroquinoline-*n*-oxide (4-NQO) (Sigma-Aldrich, China). 4NQO was the positive control without metabolic activation (PBS) and 2AA with metabolic activation (S9).

#### 5.3.2. SOS/Umu Test

The SOS/umu test was performed according to the method proposed by [62,63], with some modifications.

The test strain *Salmonella typhimurium* 1535/pSK1002 (German Collection for microorganisms and cell cultures (DSMZ)) was thawed from stock (−135 °C in TGA medium containing 10% of DMSO as cryoprotective agent) and 0.5 mL of bacteria were suspended in 100 mL TGA medium supplemented with ampicillin (50 µg/mL). The bacteria culture was incubated overnight at 37 °C with slight orbital shaking (155 rpm) from 15–17 h until an optimal orbital density (OD_600_ from 0.5 to 1.5) was reached. Then, the overnight culture was diluted with fresh TGA medium (not supplemented with ampicillin) and incubated for 2 h at 37 °C with orbital shaking (155 rpm), in order to obtain a log-phase bacterial growth culture (OD_600_ from 0.15 to 0.4). The test was performed in the absence (PBS) and presence of an external metabolic activation system (10% of rat S9 mix, prepared from S9 SD rat liver Aroclor KCl frozen, Trinova, Germany) in order to also determine the possible genotoxic effects of any metabolite. In each test performed, negative and positive controls were included, DMSO (or water for FB1) was used as solvent control (negative control), and 4-NQO and 2-AA were used as positive controls in the absence and presence of S9 mix, respectively (see maximum concentrations used in Section 5.3.1)

The test procedure was as follows: first, mycotoxins were dissolved at their respective maximum concentrations (see Section 5.3.1). Then, 11 serial ½ dilutions in DMSO (or water for FB1) of mycotoxins and positive controls were prepared in a 96-well plate (plate A). The final volume in each well was 10 µL. The wells on the last row of the plate contained the negative control (DMSO or water).

Afterwards, 70 µL water was added to each well. At this point each well was checked in order to detect any precipitation of the mycotoxins.

Thereafter, in another two 96-well plates (plates B; one for the test with S9 and the other without S9), 10 µL S9 mix or 10 µL PBS, respectively, were added, followed by 25 µL of the concentrations of the different mycotoxins previously prepared (plate A). Finally, 90 µL/well of exponentially growing bacteria suspension was added to both plates and incubated for 4 h at 37 °C with orbital shaking (500 rpm). After the incubation period, A_600_ was measured to evaluate toxicity (two plates B). Toxicity was calculated as follows:

(1) $$\%\;\mathrm{Survival}=\frac{A_{600}\;\mathrm{for}\;\mathrm{each}\;\mathrm{concentration}\;\mathrm{tested}\;}{\mathrm{Average}\;A_{600}\;\mathrm{for}\;\mathrm{negative}\;\mathrm{control}\;}\;\times \;100$$

Afterwards, for the determination of β-galactosidase activity, 30 µL/well of treatment plates (plate B) were transferred into two new 96-well plates (plates C) containing 150 µL/well of ONPG solution (2-nitrophenyl-β-D-galactopyranoside, Sigma-Aldrich, Switzerland): 0.9 mg/mL in B-buffer was prepared according to [63] for an enzymatic reaction. Plates C were incubated for 30 min at 28 °C with orbital shaking (500 rpm) in the dark. After the incubation period, the reaction was stopped after adding 120 µL/well of Na_2_CO_3_ (1M). Finally, A_420_ was measured and β-galactosidase activity was determined as follows (two plates C):

β-galactosidase activity relative units (RU):

(2) $$\mathrm{RU}:=\;\frac{A_{420}\;\mathrm{for}\;\mathrm{each}\;\mathrm{concentration}\;\mathrm{tested}\;}{\;A_{600}\;\mathrm{for}\;\mathrm{each}\;\mathrm{concentration}\;\mathrm{tested}\;}$$

Additionally, induction factor (IF)

(3) $$\mathrm{IF}=\frac{\mathrm{RU}\;\mathrm{for}\;\mathrm{each}\;\mathrm{concentration}\;\mathrm{tested}\;}{\;\mathrm{Average}\;\mathrm{RU}\;\mathrm{for}\;\mathrm{negative}\;\mathrm{control}\;}$$

where average β-galactosidase RU for negative control:

(4) $$\mathrm{RU}:=\;\frac{\;\mathrm{Average}\;A_{420}\;\mathrm{for}\;\mathrm{negative}\;\mathrm{control}\;}{\;\mathrm{Average}\;A_{600}\;\mathrm{for}\;\mathrm{negative}\;\mathrm{control}\;}$$

## Figures and Tables

**Table 1:** Carcinogenicity (IARC classification) and mutagenicity data (Ames and SOS/umu tests) of mycotoxins ^a^.

Mycotoxin	IARC Classification (Year)	Ames Test	SOS Test
Aflatoxin B1	Group 1 (2012) [8]	Positive [20,21,22,23,24,25,26,27,28,29]	Positive [30,31,32,33,34,35,36,37,38,39] Negative [40]
Sterigmatocystin	Group 2B (1976) [41]	Positive [21,23,24,25,26,42]	Positive [32,33,37,38,43] Negative [40]
Nivalenol	Group 3 (1993) [44]	No data	No data
Deoxynivalenol	Group 3 (1993) [44]	Negative [25,45,46]	Negative [45]
3-Acetyldeoxynivalenol	No classification	Negative [25]	No data
15-Acetyldeoxynivalenol	No classification	No data	No data
Fusarenon-X	No clasiffication	Negative [42]	No data
T-2 toxin	Group 3 (1993) [44]	Negative [24,25,42]	Negative [30,33]
HT-2 toxin	No classification	No data	No data
Ochratoxin A	Group 2B (1993) [44]	Negative [24,26,47,48,49] Positive [50]	Negative: [32,38] Weak positive [33] Positive [51]
Zearalenone	Group 3 (1993) [44]	Negative [24,25,52]	Negative [30,31,32,33,38] Positive [53]
Fumonisin B1	Group 2B (2002) [54]	Negative [45,55,56]	Negative [45]

^a^ The most recent International Agency for Research on Cancer (IARC) classification for each mycotoxin is shown. To retrieve results regarding Ames Test and SOS/umu, the following search strategy was followed: (a) Pubmed search *“[name of each individual mycotoxin]* AND *(ames* OR *sos* OR *“reverse mutation”)*. No filters were used; (b) Pubmed and Scifinder search: *mycotoxin* AND *(sos chromotest* OR *sos umu test* OR *sos spot test OR sos microplate).* No filters were used. Only articles presenting results of Ames Test (at least one *Salmonella* strain) or SOS tests of each individual mycotoxins were included for data extraction.

**Table 2:** DEREK Nexus predictions for mutagenicity of analyzed mycotoxins (alerts are shown as displayed in DEREK Nexus^®^).

Ref	Mutagenicity In Vitro				Mutagenicity In Vivo		Chromosome Damage (Mammal)				Non-Specific Genotoxicity In Vitro ^d^	
	Bacterium		Mammal		Mammal		In Vitro		In Vivo			
	Alert Ref ^a^	Prediction	Alert Ref ^a^	Prediction	Alert Ref ^a^	Prediction	Alert Ref ^a^	Prediction	Alert Ref ^a^	Prediction	Alert Ref ^a^	Prediction
AFB1	201	PLAUSIBLE	306	PLAUSIBLE	201	EQUIVOCAL	306, 309	PLAUSIBLE	- ^b^	- ^b^	306	PLAUSIBLE
STER	201	PLAUSIBLE	306	PLAUSIBLE	201	EQUIVOCAL	306	PLAUSIBLE	- ^b^	- ^b^	306	PLAUSIBLE
NIV	019	PLAUSIBLE	- ^b^	- ^b^	019	PLAUSIBLE	019, 309	PLAUSIBLE	019	PLAUSIBLE	- ^b^	- ^b^
DON	019	PLAUSIBLE	- ^b^	- ^b^	019	PLAUSIBLE	019, 309	PLAUSIBLE	019	PLAUSIBLE	- ^b^	- ^b^
3ADON	019	PLAUSIBLE	- ^b^	- ^b^	019	PLAUSIBLE	019, 309	PLAUSIBLE	019	PLAUSIBLE	- ^b^	- ^b^
15ADON	019	PLAUSIBLE	- ^b^	- ^b^	019	PLAUSIBLE	019, 309	PLAUSIBLE	019	PLAUSIBLE	- ^b^	- ^b^
F-X	019	PLAUSIBLE	- ^b^	- ^b^	019	PLAUSIBLE	019, 309	PLAUSIBLE	019	PLAUSIBLE	- ^b^	- ^b^
T-2	019	PLAUSIBLE	- ^b^	- ^b^	019	PLAUSIBLE	019	PLAUSIBLE	019	PLAUSIBLE	- ^b^	- ^b^
HT-2	019	PLAUSIBLE	- ^b^	- ^b^	019	PLAUSIBLE	019	PLAUSIBLE	019	PLAUSIBLE	- ^b^	- ^b^
OTA	(*) ^c^	INACTIVE	- ^b^	- ^b^	- ^b^	- ^b^	- ^b^	- ^b^	- ^b^	- ^b^	- ^b^	- ^b^
ZEA	(*) ^c^	INACTIVE	- ^b^	- ^b^	- ^b^	- ^b^	- ^b^	- ^b^	- ^b^	- ^b^	- ^b^	- ^b^
FB1	(*) ^c^	INACTIVE	- ^b^	- ^b^	- ^b^	- ^b^	- ^b^	- ^b^	- ^b^	- ^b^	- ^b^	- ^b^

^a^ alert: 019 = epoxide; 201 = bisfuranoid mycotoxin or analogue; 306 = alkyl aldehyde or precursors; 309 = alfa, beta-unsaturated ketone. ^b^ - = no prediction. ^c^ (*).The query structure does not match any structural alerts or examples for (bacterial in vitro) mutagenicity in DEREK. Additionally, the query structure does not contain any unclassified or misclassified features and is consequently predicted to be inactive in the bacterial in vitro (Ames) mutagenicity test. ^d^ No predictions for any of the mycotoxins were obtained for non-specific genotoxicity in vivo.

**Table 3:** VEGA QSAR© mutagenicity outcomes for analyzed mycotoxins.

Mycotoxin	VEGA Tool	Exp ^a^	Pdtion ^b^	SA/SM ^c^
AFB1	CAESAR	M	M	-
	SarPy/IRFMN	M	M	SM: 35, 141, 154, 158, 177, 187, 196
	ISS	M	M	SA:30
	KNN/Racross	M	M	
STER	CAESAR	M	M	-
	SarPy/IRFMN	M	M	SM: 35, 59, 84
	ISS	M	M	SA:59
	KNN/Racross	-	M	
NIV	CAESAR	NM	suspect M	SA:7
	SarPy/IRFMN	NM	M	SM: 92, 97, 113, 162, 163, 169, 177, 182, 186
	ISS	-	M	SA: 7, 10
	KNN/Racross	NM	NM	
DON	CAESAR	NM	suspect M	SA: 7
	SarPy/IRFMN	NM	M	SM: 92, 97, 113, 162, 163, 169, 177, 182, 186
	ISS	-	M	SA: 7, 10
	KNN/Racross	NM	NM	
3ADON	CAESAR	-	suspect M	SA: 7
	SarPy/IRFMN	-	M	SM: 92, 97, 113, 162, 163, 169, 177, 182, 186, 188
	ISS	-	M	SA: 7, 10
	KNN/Racross	-	NM	
15ADON	CAESAR	-	suspect M	SA: 7
	SarPy/IRFMN	-	M	SM: 92, 97, 113, 123, 162, 163, 169, 177, 182, 186, 188
	ISS	-	M	SA: 7, 10
	KNN/Racross	-	NM	
F-X	CAESAR	NM	suspect M	SA: 7
	SarPy/IRFMN	NM	M	SM: 92, 97, 113, 162, 163, 169, 177, 182, 186, 188
	ISS	-	M	SA: 7, 10
	KNN/Racross	-	NM	
T-2	CAESAR	NM	suspect M	SA: 7
	SarPy/IRFMN	NM	M	SM: 92, 97, 113, 162, 163, 169, 178, 182, 186, 188
	ISS	-	M	SA:7
	KNN/Racross	-	NM	
HT-2	CAESAR	-	suspect M	SA:7
	SarPy/IRFMN	-	M	SM: 92, 97, 113, 123, 162, 163, 169, 178, 182, 186, 188
	ISS	-	M	SA:7
	KNN/Racross	-	NM	
OTA	CAESAR	-	NM	-
	SarPy/IRFMN	-	NM	SM: 146, 170, 189, 190
	ISS	NM	NM	-
	KNN/Racross	NM	NM	
ZEA	CAESAR	NM	NM	-
	SarPy/IRFMN	NM	NM	SM: 146, 163, 170, 177, 189
	ISS	NM	NM	-
	KNN/Racross	NM	NM	
FB1	CAESAR	-	NM	-
	SarPy/IRFMN	-	NM	SM: 121, 137, 157, 163, 169, 178, 182, 188
	ISS	NM	NM	-
	KNN/Racross	NM	NM	

^a^ Exp = experimental data; ^b^ Pdtion = prediction, non-mutagenic (NM), mutagenic (M) and suspected to be mutagenic (suspect M). ^c^ SA: structural alert; SM: substructure (SMART) related with mutagenicity (1–112) or non- mutagenicity (number higher than 112); see Supplementary Material for details.

**Table 4:** VEGA QSAR© prediction quality outcomes for each mycotoxin.

Ref	VEGA Tool	Prediction Quality VEGA Models Outcomes ^a^								
		AD	ADI	Reliability	CONSENSUS	Sim	Acc	Cc	DRC	ACF
AFB1	CAESAR	INTO	1	good	M	1	1	1	True	1
	SarPy/IRFMN	INTO	1	good		1	1	1		1
	ISS	INTO	1	good		1	1	1		1
	KNN/Racross	INTO	1	good		1	1	1		1
STER	CAESAR	INTO	1	good	M	1	1	1	True	1
	SarPy/IRFMN	INTO	1	good		1	1	1		1
	ISS	INTO	1	good		1	1	1		1
	KNN/Racross	could out	0.76	moderate		0.963	**0.49**	0.75		1
NIV	CAESAR	OUT	**0**	low	NM	1	**0**	**0**	True	1
	SarPy/IRFMN	OUT	**0**	low		1	**0**	**0**		1
	ISS	OUT	**0**	low		0.80	0.51	0.51		1
	KNN/Racross	INTO	1	good		1	1	1		1
DON	CAESAR	OUT	**0**	low	NM	1	**0**	**0**	True	1
	SarPy/IRFMN	OUT	**0**	low		1	**0**	**0**		1
	ISS	OUT	**0.64**	low		0.80	0.51	0.51		1
	KNN/Racross	INTO	1	good		1	1	1		1
3ADON	CAESAR	OUT	**0**	low	NM	0.96	**0**	**0**	True	1
	SarPy/IRFMN	OUT	**0**	low		0.96	**0**	**0**		1
	ISS	OUT	**0.65**	low		0.82	0.51	0.51		1
	KNN/Racross	INTO	0.97	good		0.94	1	1		1
15ADON	CAESAR	OUT	**0**	low	NM	0.96	**0**	**0**	True	1
	SarPy/IRFMN	OUT	**0**	low		0.96	**0**	**0**		1
	ISS	OUT	**0.64**	low		0.82	0.51	0.51		1
	KNN/Racross	INTO	0.97	good		0.94	1	1		1
F-X	CAESAR	OUT	**0**	low	NM	1	**0**	**0**	True	1
	SarPy/IRFMN	OUT	**0**	low		1	**0**	**0**		1
	ISS	could out	0.90	moderate		1	1	1		1
	KNN/Racross	INTO	0.97	good		0.93	1	1		1
T-2	CAESAR	OUT	**0**	low	NM	1	**0**	**0**	True	1
	SarPy/IRFMN	OUT	**0**	low		1	**0**	**0**		1
	ISS	could out	0.86	moderate		0.78	1	1		1
	KNN/Racross	could out	0.78	moderate		0.854	1	0.52		1
HT-2	CAESAR	OUT	**0**	low	M	0.935	**0**	**0**	True	1
	SarPy/IRFMN	OUT	**0**	low		0.935	**0**	**0**		1
	ISS	could out	0.89	moderate		0.8	1	1		1
	KNN/Racross	could out	0.87	moderate		0.877	1	0.75		1
OTA	CAESAR	could out	0.72	moderate	NM	0.83	1	**0.38**	True	1
	SarPy/IRFMN	could out	0.72	moderate		0.83	1	**0.38**		1
	ISS	INTO	1	good		1	1	1		1
	KNN/Racross	INTO	1	good		1	1	1		1
ZEA	CAESAR	INTO	1	good	NM	1	1	1	True	1
	SarPy/IRFMN	INTO	1	good		1	1	1		1
	ISS	INTO	1	good		1	1	1		1
	KNN/Racross	INTO	1	good		1	1	1		1
FB1	CAESAR	could out	0.88	moderate	NM	0.908	1	0.71	True	1
	SarPy/IRFMN	could out	0.80	moderate		0.908	0.71	0.71		1
	ISS	INTO	1	good		1	1	1		1
	KNN/Racross	INTO	1	good		1	1	1		1

^a^ AD: applicability of domain; ADI: applicability of domain index; Sim: similarity; Acc: accuracy; Cc: concordance; DRC: descriptors range check; ACF: atom-centred fragments; NM: non-mutagenic; M: mutagenic; suspect M: suspected to be mutagenic. Soft grey: no optimal assessment; numbers in bold and strong grey: bad assessment. Results are expressed in a quantitative way (0 to 1), except for AD (into, out, could out) and for DRC (true, false). Reliability of the prediction, based on the overall results obtained, is expressed as good, low or moderate.

## Data Availability

Data is contained within the article.

## Supplementary Materials

The following are available online at https://www.mdpi.com/article/10.3390/toxins13100734/s1, Table S1, Descriptors calculated for analyzed mycotoxins. Figure S1: Results from SOS/umu test with (dark grey) or without S9 (light grey) activation for NIV, 3-ADON, 15-ADON, T-2 and HT-2 toxins at the highest soluble concentration (4000 µg/mL), Table S2: Structural alerts for CAESAR Model, Table S3: Structural alerts for SarPy/IRFMN Model, Table S4: Structural alerts for ISS Model.
