# Supplementary material for: Prioritization of Mycotoxins Based on Their Genotoxic Potential with an In Silico-In Vitro Strategy
Source: Toxins (Basel). 2021 Oct 19;13(10):734. doi: 10.3390/toxins13100734 (PMC8540412; doi:10.3390/toxins13100734)
Supplement: Supplementary file 1 [file toxins-13-00734-s001.zip › toxins-1339898-supplementary.pdf]

toxins

Supplementary Information

Prioritization of Mycotoxins Based on Their Genotoxic Potential With an *In Silico-In Vitro* Strategy

Maria Alonso-Jauregui [1], María Font [2,3], Elena González-Peñas [2], Adela López de Cerain [1,3] and Ariane Vettorazzi [1,3,*]

[1] Department of Pharmacology and Toxicology, Research Group MITOX, School of Pharmacy and Nutrition, Universidad de Navarra, 31008 Pamplona, Spain; malonso.17@alumni.unav.es (M.A.-J.); acerain@unav.es (A.L.d.C)
[2] Department of Pharmaceutical Technology and Chemistry, Research Group MITOX, School of Pharmacy and Nutrition, Universidad de Navarra, 31008 Pamplona, Spain; mfont@unav.es (M.F.); mgpenas@unav.es (E.G.-P.)
[3] IdiSNA, Navarra Institute for Health Research, 31008 Pamplona, Spain
* Correspondence: avettora@unav.es

Section 1. Molecular Modelling (Structural Analysis and Preliminary SAR)

Instrumental

The calculations were performed on SGI Virtu VS100 workstation provided with MOE2019.01.02 software package [90].

Molecular Modelling (Structural Analysis and Preliminary SAR)

The 3D models of the studied compounds were constructed according to the initial configuration, in gas phase, using atoms from the corresponding module of the MOE2019 and using the implemented MMFF94x Force [1–3]. A preliminary optimization Energy Minimize general strategy with a root mean square gradient, RMS, of 0.001 kcal/mol/Å^2 as completion criterion. Restraints and constraints were not applied. The first minimized conformations obtained were considered as the starting conformations for the conformational analysis carried out through a systematic search strategy with an rms gradient of 1 × 10^{-3} kcal/mol/ Å^2, an energy window of 5–10 kcal, and a maximum conformation number of 100–200. The applied protocol can be summed up as follows: (a) Initial construction of the model and first minimization by application of the minimize protocol (steepest descent algorithm with a convergence criterion of 1 × 10^{-3}). (b) Application of the routine for conformation generation (first: conjugate-gradient minimization in torsion space; second: conjugate-gradient minimization in Cartesian space; third: Quasi-Newton minimization in Cartesian space, rms = 1 × 10^{-3}). (c) Elimination of the conformations whose relative energy is greater than 5–10 kcal/mol at a global minimum. (d) Analysis of conformational trajectory and selection of representative lowest energy conformations for each analysed compound.

The descriptors were calculated for the selected representative lowest energy conformation.

Results of The Preliminary SAR: Some ADME Descriptors

In order to obtain insight regarding the effects that the aforementioned structural modifications can have on ADME profiles some descriptors have been calculated (Table 2): i) logP that provides information on the lipophilicity of the structure, ii) Topological Polar Surface Area (TPSA) which informs about the distribution and accessibility of the polar areas of the molecule, iii) van der Waals area and iv) volume as descriptors related with steric character; and the v) hydrogen bond acceptors and donors profile, calculated by means of the number of acceptor and donor moieties detected in the analysed molecules.

Regarding AFB1 and STER, the structural variation of rings that decorate the furo[2,3-b]benzo furan common scaffold, namely the 3,4,5,6-tetrahydrocyclopenta[c]pyran-1,7-dione in AFB1 to 5-hydroxychroman-4-one in STER, implies an increase in lipophilicity and size linked to the cycle expansion (from 5 C to 6 C) and the aromaticity of the new ring. An increase in the TPSA value is also detected which must be related to the different accessibility to the polar zones that entails the new distribution of the polar elements, and the change from an oxo group to hydroxyl, along with the expansion of the cycle that contains it. The number of acceptors is maintained but STER has a donor when disposing of the hydroxyl.

With regard to 1,5-dimethylspiro[8-oxatricyclo[7.2.1.02,7]dodec-5-ene-12,2'-oxirane] derivatives, and taking NIV as reference, the structural variations implies a notable modification of lipophilicity. In fact, NIV has a polar character with a logP theoretical value = -2.164 whereas T-2 toxin is markedly more lipophilic with logP = 1.765. Loss of a hydroxyl between NIV and DON implies lower values for logP, TPSA and smaller values for steric descriptors. The acceptor-donor profile shows a decrease in both acceptor and donor related with the aforementioned loss of OH.

The presence of an acetyl group decorating the common scaffold and placed at different location, which leads to 3-ADON, 15-ADON and F-X, exemplifies the repercussion that the introduction of the same structural element has on the descriptors and the related theoretical properties. Thus, it can be seen how when the acetyl moiety, which in all cases leads to an increase in the values of the steric descriptors considered, if it is introduced in position 3 or 15 (3ADON or 15ADON) or in position 2 (F-X), the molecule has a different lipophilic character. Likewise, the distribution and accessibility to the new polar elements introduced is different, increasing the value of TPSA especially for F-X.

The structural variations that lead to T-2 and HT-2, namely the presence of an methylacetyl moiety and the 3-methylbutanoate chain implies a notheworty increase of lipophilic character, specially for T-2. The variation in the number of acceptor/donor elements, shows the different theoretical profile of hydrogen bonding for these structures.

With respect to the third structural group, the three structures studied are significantly more lipophilic, especially ZEA with a logP = 3.372. FB1 has significantly higher surface and volume values than those of the other analyzed structures. Its TPSA is also high and it presents a number of acceptors and donors that exceed in both cases the values recommended by the Lipinski rule[3]. The high conformational freedom of the molecule is also notable.

Table S1. Descriptors calculated for analysed mycotoxins.

Mycotoxin	logP(o/w)	TPSA [a]	vdw_area [b]	vdw_vol [c]	a_acc [d]	a_don [e]
**AFB1**	1.216	71.06	232.3935	339.7961	5	0
**STER**	2.047	74.22	248.2020	358.3236	5	1
**NIV**	−2.164	119.75	244.5441	310.6544	7	4
**DON**	−1.149	99.52	238.3272	303.5739	6	3
**3ADON**	−0.560	105.59	284.9479	351.2902	6	2
**15ADON**	−0.560	105.59	284.9479	351.2902	6	2
**F-X**	−1.575	125.82	291.1648	358.3707	7	3
**T-2**	1.765	120.89	425.6148	502.3053	6	1
**HT-2**	1.176	114.82	378.9941	454.5890	6	2
**OTA**	3.331	112.93	340.8163	431.9343	5	4
**FB1**	2.444	288.51	684.1500	761.1516	14	12
**ZEA**	3.372	83.83	297.2245	364.4836	4	2

[a] TPSA = Topological Polar Surface area, in Å^2. [b] van der Waals area, in Å^2. [c] van der Waals volume, in Å^3. [d] a_acc = number of hydrogen bond acceptors. [e] a_don = number of hydrogen bond donors.

Section 2

Table S2. Structural alerts for CAESAR Model.

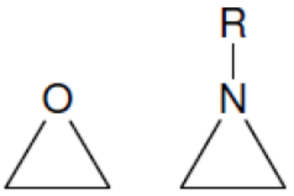 **R = any atom/group**
SA7 Epoxides and aziridines

Table S3. Structural alerts for SarPy/IRFMN Model.

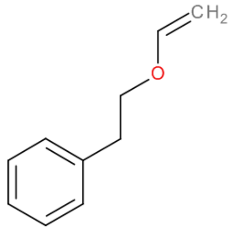	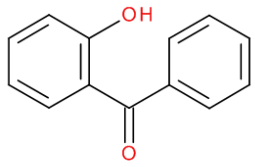	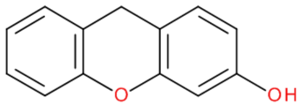
SM 35: C(c1ccccc1)COC=C	SM 59: O=C(c1ccccc1O)c2ccccc2	SM 84: Oc1ccc2Cc3ccccc3Oc2c1
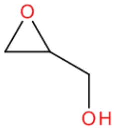	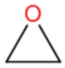	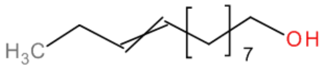
SM 92: OCC1OC1	SM 97: C1OC1	SM 113: C(O)CCCCCCCC=CCC
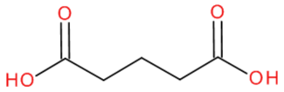	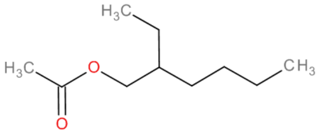	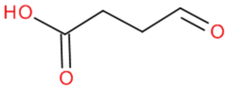
SM 121: C(=O)(C(CCC(=O)O))O	SM 123: CC(=O)OCC(CC)CCCC	SM 137: C(=O)(CCC(=O)O)
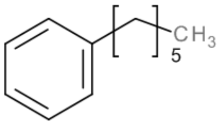	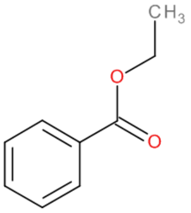	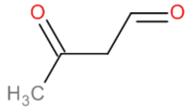
SM 141: c1(CCCCCC)ccccc1	SM 146: c1(C(=O)OCC)ccccc1	SM 154: C(=O)CC(=O)C
H_3C-$(CH_2)_{10}$-CH_3	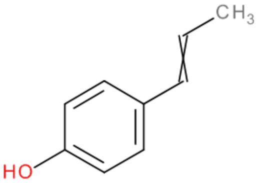	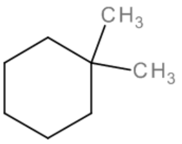
SM 157: CCCCCCCCCCCC	SM 158: c1(c(ccc(c1)C=CC)O)	SM 162: C1CC(CC(C1))(C)C

SarPy/IRFMN Model; rules for mutagenicity (1-113) and non-mutagenicity (>113), expressed as SMARTS strings.

Table S3. (Cont).

H_3C-$(CH_2)_5$-CH_3	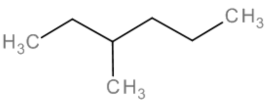	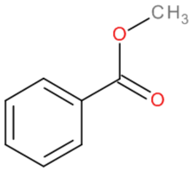
SM 163: CCCCCCC	SM 169: CC(CC)CCC	SM 170: C(=O)(c1ccccc1)OC
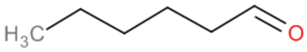	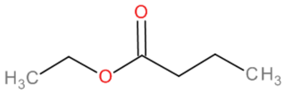	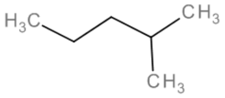
SM 177: C(=O)CCCCC	SM 178: C(=O)(CC(C))OCC	SM 182: CCCC(C)C
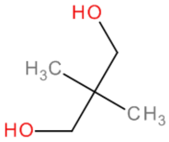	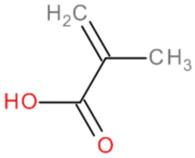	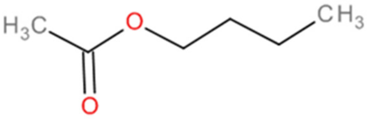
SM 186: OCC(CO)(C)C	SM 187: OC(=O)C(=C)C	SM 188: C(=O)(C)OCCCC
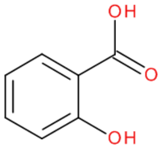	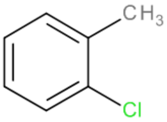	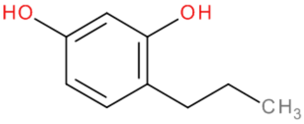
SM 189: c1(C(=O)O)c(ccc(c1))O	SM 190: c1(c(Cl)cccc1)C	SM 196: c1(cc(ccc1CCC)O)O

Table S4. Structural alerts for ISS Model.

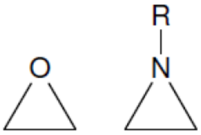 **R = any atom/group**	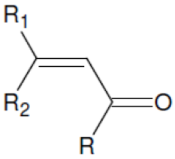 **R_1 and R_2 = any atom/group, except alkyl chains with C > 5 or aromatic rings. R = any atom/group, except OH, O^-**	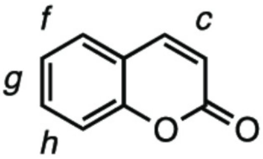
SA7 Epoxides and aziridines	SA10 alfa, beta unsaturated carbonyls	SA30 Coumarins and Furocoumarins[a]
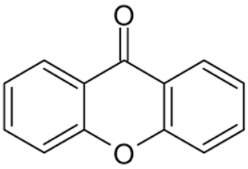 **(a)**	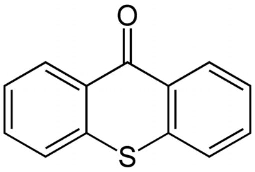 **(b)**	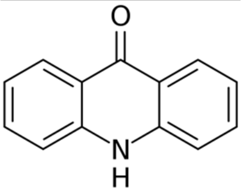 **(c)**
SA59 **(a)** Xanthones, **(b)** Thioxanthones, **(c)** Acridones [b]		

[a] Furocoumarin show a furano ring condensed with the alfa-benzopirone ring (c, f, g or h side), the common scaffold for coumarins and furocpumarins. [b] DNA intercalating agents are defined as those compounds that are able to insert partially or completely between adjacent DNA base pairs. Fused polycyclic chemicals are classical members of this class of compounds.

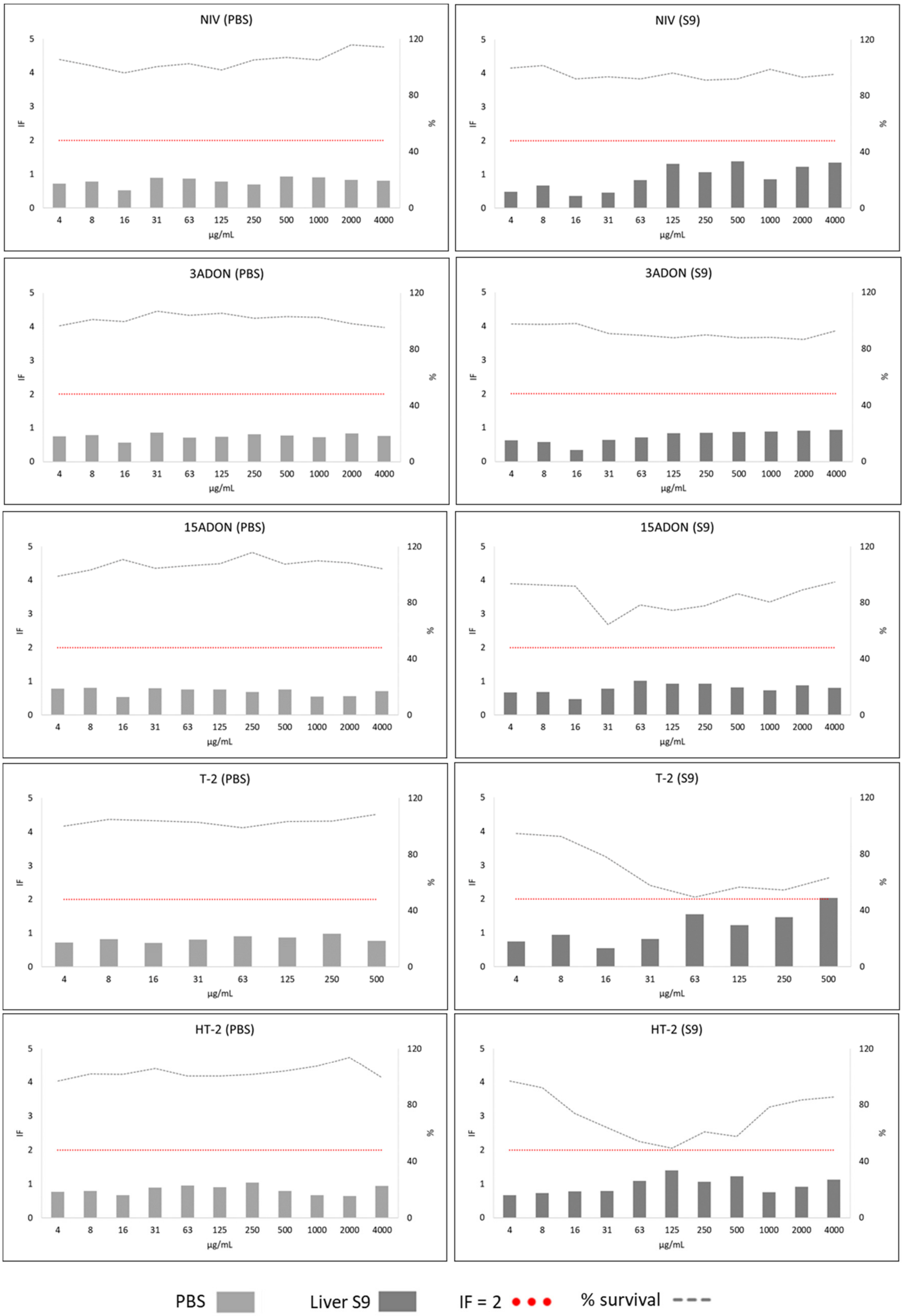

Figure S1. Results from SOS/umu test with (dark grey) o without S9 (light grey) activation for NIV, 3-ADON, 15-ADON, T-2 and HT-2 toxins at the highest soluble concentration (4000 µg/mL). T-2 toxin had precipitates at 1000, 2000 and 4000 µg/mL. Concentrations are considered non toxic if survival is >80%. A compound is considered genotoxic if the induction factor is ≥2 at non toxic concentration for the bacteria in any of the concentrations tested.

References

1. Halgren, T.A. Merck molecular force field. I. Basis, form, scope, parameterization, and performance of MMFF94. *J Comput. Chem*. **1996**, 17(5–6), 490–519, https://doi.org/10.1002/(SICI)1096-987X(199604)17:5/6%3C490::AID-JCC1%3E3.0.CO;2-P.
2. Halgren, T.A. Merck molecular force field. V. Extension of MMFF94 using experimental data, additional computational data, and empirical rules. *J. Comput. Chem.* **1996**, *17*, 616–641, https://doi.org/10.1002/(sici)1096-987x(199604)17:5/6<616::aid-jcc5>3.0.co;2-x.
3. Lipinski, C.A., Lombardo, F., Dominy, B.W., Feeney, P.J. Experimental and computational approaches to estimate solubility and permeability in drug discovery and development settings. *Adv. Drug Deliv. Rev.* **2001**, 46(1–3), 3–26., https://doi.org/10.1016/s0169-409x(00)00129-0.
